# A health sciences library promotes wellness with free yoga

**DOI:** 10.5195/jmla.2019.475

**Published:** 2019-01-01

**Authors:** Tallie Casucci, Donna Baluchi

**Affiliations:** J. Willard Marriott Library, University of Utah, Salt Lake City, UT, tallie.casucci@utah.edu; Spencer S. Eccles Health Sciences Library, University of Utah, Salt Lake City, UT, donna.baluchi@utah.edu

## Abstract

**Background:**

Yoga is a popular physical exercise activity with mental health benefits. Public and academic libraries have offered free yoga as their primary movement-based program.

**Case Presentation:**

In an attempt to bolster wellness and connect to the health sciences community, an academic health sciences library offered free yoga as a ten-week trial series in summer 2016. At the end of the trial series, weekly attendance and online feedback data determined that this series should continue.

**Conclusions:**

Yoga provides health professionals and students with a welcomed midday break from a stressful work environment. Additionally, by partnering with yoga instructor trainee programs, the financial burden is low for the library.

## BACKGROUND

Yoga is gaining popularity, with increased participation each year. The *2016 Yoga in America Study* conducted by *Yoga Journal* and the Yoga Alliance concluded that the number of Americans who attended a yoga class within the prior 6 months increased from 20.4 million in 2012 to more than 36 million in 2016, with another 80 million stating that they would be willing to try yoga for the first time within the next 12 months [1]. Additionally, there is a growing perception that yoga is for everyone, including men and older individuals [1]. The clinical benefits of practicing yoga include reduced stress and anxiety, improved mood, reduced heart rate, less chronic back pain, and decreased blood pressure [2, 3]. Yoga is one of the top 10 integrative health practices among adults in the United States [4].

In 2017, a survey of nearly 1,000 librarians in the United States and Canada found that yoga was the largest movement-based program in public libraries [5]. Most yoga programs in public libraries have started within the last few years [5]. Many public libraries have incorporated movement into their programming specifically to encourage healthy practices among their patrons [6]. Academic libraries have hosted yoga events at the end of semesters when students were studying for their final exams [7–9]. Other libraries have offered yoga for employees as part of employee wellness programs [10–12]. For example, the University of California San Diego Library began a yoga program for employees in 2007 “because it could be accommodated in an existing library space, required little equipment, and wouldn’t disrupt patrons” [12].

For medical libraries, yoga is a compelling activity due to their patron populations, because both health professionals and students report high burnout rates and poor personal wellness indicators [13, 14]. Burnout is a major concern for health care, as it can impact patient outcomes and safety [13, 14]. Recent literature suggests that mindfulness and exercise can help physicians and other health care professionals who are struggling with burnout and job satisfaction [15–19]. One specific preventative program offered to University of Wisconsin medical students is a curriculum that “includes several full days of exercises devoted to exploring mindful practice, compassion, enhancing self-awareness, and building resilience. Students are introduced to a variety of techniques to enhance these skills including yoga, meditation, walking, noticing, and breathing exercises” [15]. Additionally, the Texas Medical Center Library has offered pay-as-you-go yoga for library employees and anyone else at the Texas Medical Center [20].

## STUDY PURPOSE

In an attempt to bolster wellness and connect to the health sciences community, the University of Utah’s Spencer S. Eccles Health Sciences Library (Eccles Library) offered free yoga sessions as a ten-week trial series in the summer of 2016 for health campus students and employees. At the end of the trial, staff determined based on weekly attendance and online feedback data that this series should continue as a new permanent event series.

## CASE PRESENTATION

Prior to 2016, the University of Utah libraries did not offer free yoga sessions in their spaces. To explore the concept of free yoga sessions, we contacted a local yoga instructor training program [21]. Since yoga instructor trainees must complete volunteer hours to earn their certificates, we believed that this training program would benefit from partnering with the library because it would provide another location for accruing volunteer hours. The yoga instructor training program immediately agreed to the partnership and found us an instructor.

Although other yoga programs were offered through the university’s College of Health, Continuing Education, and Campus Recreation [22–24], our library-based yoga series was unique in that it was completely free for participants. Also, our yoga program provided a more convenient location for individuals on the university’s health campus, compared with the other yoga programs that were located on the main campus and, thus, required about twenty to thirty minutes of travel each way. Furthermore, free yoga sessions complemented our library’s existing wellness-related offerings, including free art exhibits, chair massages, guest lectures, spoken-word café events, therapy dogs, treadmill desks, puzzles, and coloring supplies [25].

The library’s expenses to support the free yoga series were minimal, with the only ongoing cost being parking expenses for the yoga instructor (~$80.00 per year). Additionally, we made a one-time purchase of 3 yoga mats to support the trial yoga series, with a total budget of $35.00. The 3 mats were uniquely barcoded, added to the library catalog, and provided for participants’ convenience. We also anticipated that the number of mat check-outs would serve as a data source for evaluating the success of the trial series.

To add another incentive to participate in the trial yoga series in addition to the removal of financial barriers, we contacted the facilitators of the WellU and WellnessNow programs that provide insurance discounts to university employees [26, 27]. The WellU program agreed to provide wellness activity credit for individuals who attended six yoga sessions.

We advertised the free yoga sessions via normal library communication channels, including university calendars, blog posts [28, 29], social media, printed flyers, targeted emails, and announcements through other university groups. The yoga sessions were scheduled to be held in the History of Medicine room for the entire trial series. After completely filling the room with twenty-three people during the first session, most subsequent sessions were moved outside under trees on the grass.

Wednesday at noon was chosen as the day and time for the series, which avoided other library-hosted events and recurring health campus events, such as grand round lectures. Additionally, the primary organizer could dedicate Wednesday lunchtimes to the event series. For the trial series, each yoga session lasted an hour and was taught by the same yoga instructor. If the instructor was unavailable for a yoga session, they were expected to find a substitute instructor. At least one library employee was available to assist the yoga instructor each week. For the trial series, the library employee would set up the space when inside, move the advertisement café sign by the space, take attendance, facilitate the WellU sign-up, and make announcements.

An online Springshare LibWizard survey was created and shared at the beginning of the advertising process. The survey contained five mostly free-text response questions:

1. Did you attend? (Yes/No/Not Yet)
2. What did you enjoy about this event? What should we keep?
3. What would you add to the program?
4. What would you drop or change?
5. Would you attend future “Free Yoga at the Eccles Library” events”?

Midway through the series, a short uniform resource locator (URL) to the survey was printed on small slips of paper and given to participants. As this was a trial series, we and the yoga instructor emphasized to participants the importance of this feedback to the potential continuation of the yoga series.

**Figure 1:**
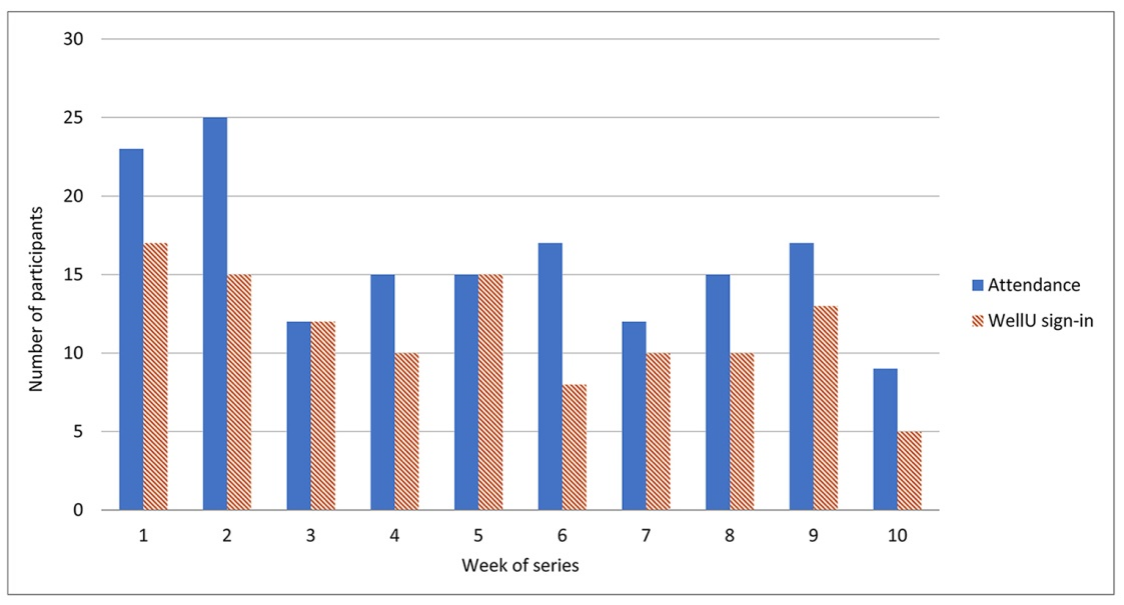
Trial series attendance and WellU sign-in participation

After the end of the ten-week trial yoga series, we reviewed the attendance numbers, number of yoga mat check-outs, and participant feedback. The participants for each yoga session were mostly university employees and a few students. Attendance averaged sixteen people each week, ranging from nine to twenty-five participants (Figure 1). Throughout the series, thirty-nine unique individuals signed-in for WellU credit and eight people received credit for attending six or more yoga sessions. Both attendance and WellU sign-in numbers dropped around federal and state holidays in weeks seven and ten.

The yoga mat check-out data were disregarded due to inconsistencies because the circulation staff did not know that the yoga mats were uniquely cataloged. If all of the yoga mats were checked out, the instructor would provide a mat to participants. If we were still out of mats, participants would use a blanket or nothing. Some participants permanently stored their “work yoga mats” in their offices.

Twelve people completed the feedback survey. Following Strauss and Corbin’s grounded theory of analyzing qualitative data [30], 5 thematic categories emerged from the survey responses of 9 individuals who attended 1 or more yoga sessions. The responses of 3 nonattendees who completed the survey were considered separately. This represents an estimated <20% response rate among the yoga participants.

The first category had thirteen comments. This “class” category included aspects of the class itself, such as the instructor, participants, and free yoga opportunity. One participant summarized this category by stating, “It was free. I really enjoyed the instructor and all the other participants they didn’t judge me.” This category also included comments about making the class a priority during their work-week, as one participant stated, “I work through my lunch time on other days so that I can attend this. I look forward to it.”

The second category had twelve comments. This “work break” category included those who enjoyed the convenient midday break and escape from the office. The work break category included comments such as “It is the perfect opportunity to break away from work,” “So so important to have the chance to do this at work at lunch hour,” and “I really need this in the middle of the day.”

The third category also had twelve comments. This “health” category covered both mental and physical benefits of yoga. As one participant noted, “Yoga is something that is so good for the mind and body and I think it is lovely that we get to participate in it here.” Others enjoyed the exercise and stated, “it…rejuvenates me.”

The fourth category included seven comments about “being outside.” This “being outside” category included positive and negative comments about being outside in nature. Two participants described being outside as “amazing.” Another participant stated, “I would say being outside is nice and everything but the grass is bumpy and the tree roots are really close to the surface. it makes it a little bit harder to balance.”

Finally, the last category included seven comments. This “future” category included comments from those who wanted the yoga series to continue and those who wanted yoga offered on other days and times. Some examples from this category included: “Love this, please keep it available,” “two days a week?,” and “[Do] not end it! Please keep it going!”

The three nonattendee survey participants made comments that fit into the aforementioned “class” and “future” categories. The nonattendees appreciated the free aspect of the class. In the future category, they recommended another time, ideally “earlier times for those who have a 9–5 job.” Also, in the future category, a nonattendee recommended another location.

Based upon attendance and feedback data, the Eccles Library faculty committee supported our proposal to continue offering free yoga sessions at the library, using a more flexible space and experimenting with different times to address the participants’ suggestions for yoga outside of the 9:00 a.m.–5:00 p.m. work schedule. Eccles Library faculty committee members were particularly impressed by the attendance numbers, as attendance at most library events is typically in the single digits. Additionally, the library faculty committee recognized that free yoga exposed new community members to the library’s resources, services, events, and personnel. In some cases, attendees of the yoga sessions attended other library events and scheduled meetings with librarians for help on research projects.

After the trial series, the permanent yoga series utilized a more flexible space in the basement that contains light furniture on wheels that can be easily moved before and after the yoga sessions. When available, library staff assisted with setting up the space. As one participant commented, “I loved the calm atmosphere…The room was pretty secluded and it was easy to focus and relax there.” Additionally, this space resolved concerns about the necessity of an indoor space during the winter months.

Based upon feedback from the trial series, yoga sessions outside of the 9:00 a.m.–5:00 p.m. work schedule were tested over the next year and a half (Table 1). After testing both morning and evening times, the organizers decided the best time was noon. Attendance numbers were largest on Wednesdays at noon, despite the additional times offered and suggestions for yoga outside of the 9:00 a.m.–5:00 p.m. work schedule. Consistent with that the second largest qualitative category from the trial series feedback survey was being a “break from work,” the convenient lunch break seemed the most compelling time for people to attend.

**Table 1:** Permanent yoga series participation

Semester	Day and time	Number of sessions	Number of participants	

			Range	Mean
Fall 2017	Wednesday noon–1:00 p.m.	14	2–13	7.3
	Thursday 7:00 a.m.–8:00 a.m.	15	0–8	3.9
Spring 2017	Monday 5:15 p.m.–6:05 p.m.	12	3–7	5.4
	Wednesday 12:05 p.m.–12:55 p.m.	14	6–17	11.1
	Friday 8:05 a.m.–8:55 a.m.	15	1–8	3.3
Summer 2017	Monday 5:15 p.m.–6:05 p.m.	3	1–7	4.9
	Wednesday 12:05 p.m.–12:55 p.m.	12	5–16	10.1
Fall 2017	Wednesday 12:05 p.m.–12:55 p.m.	11	4–16	10.3
	Friday 12:05 p.m.–12:55 p.m.	11	2–11	7.0
Spring 2018	Monday 12:05 p.m.–12:55 p.m.	1	13	13.0
	Wednesday 12:05 p.m.–12:55 p.m.	10	1–12	7.4
	Friday 12:05 p.m.–12:55 p.m.	10	4–12	10.0

Yoga session attendance decreased after the trial series. One potential reason for this decrease might be that participants prioritized attending sessions during the trial series to support the continuation of the series, which we heard in informal conversations. Another potential reason for the decline in attendance was the removal of the WellU credit due to changes in the WellU program’s fitness activity requirement. Finally, another reason for this decline in attendance might be due to class schedules changes.

In the spring of 2017, 2 logistical changes were made to the permanent yoga series. First, the yoga sessions were shortened to 50 minutes. This allowed participants to change clothes and attend other meetings. As one participant wrote, “I hate having to leave early, so a 45–50 minute class would give me more of a buffer to get to the next thing.” Second, a participant waiver was added. Although risk was minimal and there has not been an incident, university general counsel advised that “travel waivers” be a requirement for instructors and participants [31]. These waivers are good for the entire year and must be stored securely for 3 years. In 2017, 94 unique individuals completed a travel waiver.

With the increase in yoga times and required waivers, the organizer devised methods to increase the yoga instructors’ responsibilities to reduce the weekly time commitment for the organizer. The organizer created a document that outlined the logistics, responsibilities, and procedures for the yoga instructors. Once connected with the new yoga instructor, the organizer emails the document to the instructor to answer most of their questions. The document contains information, such as the location (directions, parking, and library space), participants, expectations for substitute instructors, and weekly announcements (waivers and feedback).

On the first day, the organizer orients the yoga instructor to the various areas where items are located. For example, instructors must pick up a clipboard at the front desk that contains an attendance tracking sheet and waivers for participants. After the first week, the yoga instructors can complete their preparation duties and teach without supervision. Every few weeks, the part-time front-desk employees enter attendance data into the library’s statistics database. By placing more responsibility onto the instructor, the organizer only needs to attend and observe the instructor. This was crucial to minimize the organizer’s weekly time commitment, especially as more yoga times have been added to the free yoga series.

Three questions were added to the online Springshare LibWizard feedback survey for the permanent series. The first new question, “What day(s) did you attend free yoga?,” included multiple choice options, depending upon the semester. The next two new questions sought free-text responses: “How did you hear about Free Yoga at the Eccles Library?” and “Is there anything else you want to tell us? (If you want us to follow-up with you, please provide your contact information.).” These three questions were added to the survey to assist us in understanding the provided feedback. The question regarding how participants learned about free yoga helped us focus our advertising efforts to print flyers, emails, and word of mouth.

One critical element that benefits the yoga instructor trainees is the formal feedback that the organizer writes to the local yoga instructor training program about the trainee, consisting of a formal letter that lists the instructor trainee’s strengths and one or two areas to improve. The most common areas for the instructors to improve are their transitions between flows, cueing proper technique, and time management.

Additionally, the organizer encourages the yoga instructor trainees to ask for feedback throughout the semester from the participants. The participants provide feedback to the instructors through in-person conversations and short written notes. We have found that the yoga instructor trainees who ask for and accept feedback improve much more quickly. As teachers of students and patients, the participants can offer concrete suggestions for improvement and genuinely want to help the yoga instructor trainees improve. The yoga instructors are encouraged by participants to practice teaching different styles of yoga. Most instructors start by teaching basic hatha and/or vinyasa yoga. As they develop more confidence, some instructors will teach ashtanga, modified bikram, yin, and iyengar yoga, or they will ask the participants for their preferences (yoga style, body area focus, etc.) before a session begins.

## DISCUSSION

Our permanent yoga series is most similar to yoga programs at public libraries, as it is free and available to all. As Quatrella and Blosveren stated, “the library is no longer just a place for doing homework assignments, [it is a] community center” [32]. Libraries as physical community places are still important [33]. Also, although most of our collections are digital, offering the yoga sessions in the library’s physical space is important to the community of health care students and professionals because it provides a reason to remove themselves from their typical and stressful workspaces. As one participant stated, “I enjoy taking a break from my stressful job to get some exercise and re-center.” Additionally, the library’s permanent yoga series has brought new patrons into the library. This finding corroborates Lenstra’s survey, which found that 60% of libraries believed that their yoga programs brought new users to the library [5].

Similar to fitness events in other libraries [5, 34], our yoga sessions use volunteer instructors, which significantly lowers the cost for the library. At the highest-end of reported costs, Goodson stated that the University of California San Diego Library “pays roughly $4000 per year for nearly 100 annual yoga sessions” for their free yoga program for library employees [12]. Other libraries have avoided these costs by librarians teaching [35] and completing a certified yoga instructor program [36, 37]. Finally, another library passed the cost onto their employees, who pay for the yoga sessions [20].

With our volunteer yoga instructor program, we only pay their parking fees (~$80.00 per year). Other libraries could investigate more affordable options for securing yoga instructors. For example, Lenstra has found that public libraries utilized funds from programming budgets (40%), “Friends of the Library” groups (23%), general budgets (22%), and donations (10%), or other funding models (12%) [5]. Our main campus library will start a free yoga partnership with the on-campus yoga instructor certification course in September 2018 [38]. If successful, this would be another model for other libraries to avoid costs, since the students are already on campus and need volunteer teaching hours. In fact, several of our yoga instructors were university students, so we did not need to pay for their parking, which helped lower the financial burden.

With either volunteer or paid instructors, there can be unexpected personal emergencies or illnesses. During these unexpected yoga instructor absences, we play online yoga videos, such as *DoYogaWithMe* and *Yoga with Adriene* [39, 40]. A few regular yoga attendees have verbally mentioned to library employees that they will not attend yoga if videos are shown. Although free online videos are a very affordable and easy option, our community was not interested in this option long-term.

Locating a physical open space with semi-privacy can also be a challenge for libraries. Similar to the University of California San Diego Library, we use a flexible multipurpose space [12]. Another library created a “wellness room” for their employee yoga and wellness offerings [20]. As mentioned earlier, our permanent yoga series utilizes an open group study space with lightweight furniture. The tables have wheels, which make it easier to push them against the walls, and the chairs can be stacked. Occasionally, another event will force the permanent yoga series to move into a smaller conference room. Fortunately, this is a rare occurrence, and participants have been understanding of the situation. Other libraries can consider physical spaces that offer some privacy and openness to avoid lengthy preparation.

In addition to establishing the library as a place in the medical community and being a low-cost event series, free yoga at the library also introduces a complementary and integrative health option. This is especially important as both health care students and professionals are experiencing high levels of burnout [3–19]. Mindfulness and exercise, such as yoga, can help mitigate this burnout [15–19]. Finally, yoga programs can advance medical librarianship by providing librarians the opportunity to informally network with colleagues outside of the library. Librarians can demonstrate that they care for their communities beyond just their communities’ information needs.

Health sciences professionals and students are very stressed, so a midday exercise break is an easy service that a library can provide. As a library event series, yoga is financially feasible and easy to manage. Other health sciences libraries may consider a similar series as it builds goodwill in the community. As one participant writes, “Thank you so much for offering these. They are a priceless benefit.”
